# Unraveling the functional genes present in rhizosphere microbiomes of *Solanum lycopersicum*

**DOI:** 10.7717/peerj.15432

**Published:** 2023-06-02

**Authors:** Afeez Adesina Adedayo, Ayomide Emmanuel Fadiji, Olubukola Oluranti Babalola

**Affiliations:** Faculty of Natural and Agricultural Sciences, North-West University, Potchefstroom, North West, South Africa

**Keywords:** Disease-resistant genes, PGP genes, Phytohormones, Plant rhizobiomes, Shotgun metagenomics, Tomato

## Abstract

The microbiomes living in the rhizosphere soil of the tomato plant contribute immensely to the state of health of the tomato plant alongside improving sustainable agriculture. With the aid of shotgun metagenomics sequencing, we characterized the putative functional genes (plant-growth-promoting and disease-resistant genes) produced by the microbial communities dwelling in the rhizosphere soil of healthy and powdery mildew-diseased tomato plants. The results identified twenty-one (21) plant growth promotion (PGP) genes in the microbiomes inhabiting the healthy rhizosphere (HR) which are more predomiant as compared to diseased rhizosphere (DR) that has nine (9) genes and four (4) genes in bulk soil (BR). Likewise, we identified some disease-resistant genes which include nucleotide binding genes and antimicrobial genes. Our study revealed fifteen (15) genes in HR which made it greater in comparison to DR that has three (3) genes and three (3) genes in bulk soil. Further studies should be conducted by isolating these microorganisms and introduce them to field experiments for cultivation of tomatoes.

## Introduction

The rhizosphere is the region of soil covering the plants root where the biochemistry of the soil is influenced by the roots. Plant roots produce exudates like amino acids, sugars, organic acids, high molecular weight polymers, and vitamins that support the diversity of microbes in the rhizosphere. The exudates likewise activate interaction between the plant root and the microbiomes dwelling in the soil (Olanrewaju et al., 2019). Different microbial communities have been reported to dwell in the rhizosphere of the tomato plant, among which are Proteobacteria, Bacteroidetes, and Actinobacteria were the most abundant phyla in the rhizosphere of a tomato plant (Cheng et al., 2020; Lee et al., 2016). However, some studies have reported Acidobacteria, Firmicutes, Gemmatimonadetes, and Verrucomicrobia as representative taxa in the rhizosphere of tomatoes and other crops (Adedayo, Fadiji & Babalola, 2022a; Mohan et al., 2022; Zuo et al., 2021).

Microbial species diversity is made up of microbial species richness, microbial population present, species evenness, and microbial species distribution (Lamb, Kennedy & Siciliano, 2011). The different methods used for studying microbial communities are of two types (Kirk et al., 2004). They are biochemical-based and molecular-based techniques. Microbial diversity studies comprise the diversities of microbial communities across the biotic and abiotic factors including a gradient of stress, and disturbance among others (Kirk et al., 2004). It is challenging to study microbial diversity with biochemical techniques as a result of the inability to determine the accuracy of the detection methods and unable to determine what is present in the employed samples (Váradi et al., 2017). So, scientists mostly simplify the collected data by diversity studies into discrete, numerical quantification like diversity indices (Mondini, Noorani & Pagnotta, 2009). The following are typical examples of biochemical-based procedures; plate count, community-level physiological profile (CLPP), and fatty acid methyl ester analysis. In molecular-based techniques, it is mostly employed to study the microbial diversity in soil samples. The followings are the example of methods in molecular-based techniques; guanine plus cytosine (G+C), nucleic acid reassociation and hybridization, DNA microarray and DNA hybridization, denaturing and temperature gradient gel electrophoresis (DGGE and TGGE), single strand confirmation polymorphism (SSPS), restriction fragment length polymorphism (RFLP), terminal restriction fragment length polymorphism (T-RFLP), ribosomal intergenic spacer analysis (RISA), automated ribosomal intergenic spacer analysis (ARISA).

The plant rhizobiomes are made up of various microbial communities that play specific functions in the ecosystem. These functions include recycling nutrients, inhibiting pathogens causing diseases, production of various enzymes, and organic matter mineralization that consistently improves soil nutrients (Palansooriya et al., 2019). Some rhizosphere organisms promote the development of the plant through the acquisition of nutrients, inhibition of disease invasion, and improve stress tolerance (Zolti et al., 2020). These microbes likewise solubilize phosphate and the assimilate nitrogen that is required for plant growth (Richardson et al., 2009). These beneficial plant microbial communities colonize the rhizosphere soil as a result of production of the exudates from plants’ roots (Nwachukwu, Ayangbenro & Babalola, 2021). Associations that promote the development of crop plants are regarded as a direct mechanism, however, an association that builds on inhibiting the spoilage organisms is one of the indirect mechanisms that is otherwise known to be favorable or positive interplays. The total population of endophytic bacterial communities was the same among the employed samples which showed that the soil and the average condition of weather have no effect on the entire endophytic microbial community inhabiting the sweet potatoes (Puri et al., 2019; Puri et al., 2018).

Tomato plants are often affected by various environmental stresses like low temperature, drought, salinity, and UV light, which destroy DNA, lipids, and proteins thereby decreasing plants’ genome stability, growth, and crop production (Krishna et al., 2019). The plants react to the above-mentioned stresses through the molecular pathways and changes in classes of gene expression profiles that involve those in nucleic acid metabolism. These are induced in response to the destruction of DNA by initiating a pathway of DNA repair enzymes like DNA helicases that catalyze the breakage of the hydrogen bonds holding together the two strands of DNA (Smulders et al., 2021).

Studies have emanated on the functional genes present in the microbial communities living in the soil (Fierer et al., 2012; McGuire & Treseder, 2010). Various genes produced by microorganisms living in the soil are significant for knowledge of the dynamics of important processes. These reveal how genes promote values in agricultural practices. The whole-genome sequencing has been employed in various studies of soil samples to reveal their diversity of microbial functional metagenomes (Brumfield et al., 2020; Gupta et al., 2014). The functional metagenomes are of various types depending on their activity in the soil or other samples. These genes are known to contribute to crop plant growth and promote plant health status (Ke et al., 2019).

In tomato and other crop plants, the plant root tips, buds, and stems produce chemical substances called phytohormones (Adedayo et al., 2022). Studies on rhizosphere soil have revealed how PGPR present in the rhizosphere soil of tomato plants produced functional genes that contribute to plant growth promotion and disease-resistant potential (Adedayo et al., 2022). Typical plant phytohormones include auxins, gibberellin, cytokinin, *etc*. Hormones like indole-3-acetic acid (IAA) and ACC deaminase contribute to the growth of the tomato plant and likewise assist in nitrogen fixation and solubilization of phosphorus. These elements *i.e*., N and P are the most essential elements required for promoting the growth of tomatoes and other crops (Shah et al., 2020).

Ottesen et al. (2019) have explored the combination of phages that have been used to tackle the bacteria wilt in tomatoes. However, limited studies have reported the significance of the PGP genes produced by the rhizosphere microbial communities affect the growth and health status of plants (Chukwuneme, Ayansina & Babalola, 2021; Hu et al., 2020; Stavridou et al., 2021). Although, earlier studies reported the functional genes in plant microbiomes and also discovered that most of the plant-beneficial genes have not been identified (Fadiji, Ayangbenro & Babalola, 2021b).

In this research work, we examined plant-growth-promoting (PGP) and disease-resistant functional genes resident in the rhizobiomes of diseased and healthy tomato plants and bulk soils using the shotgun metagenomics method. Unlike other metagenomic approaches, shotgun metagenomics is employed by various researchers because it gives a wide view of the functions occurring in metagenomes and the details of the total microbiomes present in employed samples (Adedayo, Fadiji & Babalola, 2022b; Ortiz-Estrada et al., 2019). Limited research has been conducted on PGP and disease-resistant genes present in the rhizosphere of tomatoes employing a shotgun metagenomic approach. Thus, our study presents the PGP genes and disease-resistant genes produced by the rhizobiomes present in the tomato rhizosphere soil using shotgun metagenomic sequencing as well as investigates how the health status of the plant affects their expression. Therefore, we envisage that PGP genes and disease-resistant genes will be abundant in HR as compared to DR.

## Survey methodology

This study revealed the potential of putative metagenomes effectively contributing to the healthy production of tomato crops as well contribute to plant growth. To ensure proper observation of the study and to investigate the study’s objectives, a comprehensive investigation of reported studies on the potential genes was employed following the method (Akinola, Ayangbenro & Babalola, 2021; Fadiji, Ayangbenro & Babalola, 2021a; Naik et al., 2008). Conducted studies results were compared and complied employing the online endnote library system. The system assists in compiling and citing the articles used in the manuscript. Yet, we surveyed the titles, abstracts, and the conclusion of the literature to determine the useful ones.

## Materials and Methods

### Soil sample collection

The rhizosphere and bulk soil used in this study were obtained in the North West region of South Africa. The rhizosphere soil was collected directly from the uprooted roots of healthy and diseased (powdery mildew) tomato plants 45 m apart from each other. The bulk soil is a natural grassland with no tomato plantation and was collected 40 m away from the tomato plantation field. The rhizosphere soils were collected from the soil located at the root of the tomato plants. A sterile soil auger was used to dig the soil to a depth of 4–15 cm deep and the soil samples were obtained into sterile zip-lock plastic bags. More of the soil attaching to the root of the tomato plant was removed by slightly shaking to lose the attached soil into the bag.

On the farmland where the soil samples were collected, we sectioned the site into three locations for both healthy and diseased tomato farm sites. In each location, we collected rhizosphere samples from five (5) tomato plants (35–45 cm tomato plant height from its root to the apex) for healthy as well as diseased. A replicate was acquired from the soil samples obtained from five tomato plants for both healthy and diseased plants and pooled together. So three replicates comprising five plants were selected for each site, in all, 15 tomato plants were employed and pooled together. From the rhizosphere of the tomato, five healthy or diseased soil was collected and pooled together to form a replicate of the healthy rhizosphere of the tomato (HR) and diseased rhizosphere of the tomato (DR). As for the bulk soil (BR), the region is 40 m away from the tomato farm but has no tomato plantation. From the three regions of the farmland, three (3) replicates were produced for the soil samples gotten from the HR, DR, and BR (Babalola, Adedayo & Fadiji, 2022). The soil samples were collected into sterile polythene bags kept in cold boxes comprising ice cubes and moved to the lab at 4 °C. Then, the collected soils were further maintained at the lower temperature of −80 °C in the cold room.

### Description of site and experimental design

The farmland where the soil samples were collected at North-West University at an altitude, of 159 m, and coordinate 25°47′19.1"S, 25°37′05.1"E; 26°019′36.9"S, 26°053′19.0"E; 25°47′17.0"S, 25°37′03.2"E. The region is the sub-Saharan part of South Africa and is known for grasslands, shrubs, and scanty tree plantations. The average degree of hotness and coldness experienced in this region varies, the temperature falls to 8 °C and lowers during the winter period and 18–36 °C in the summer period. Every year, the rainfall in this region is approximately 360 mm, slated between November and May. This experimental farm has had a history of tomatoes, chili peppers, and other vegetable plantations for several years. Furthermore, the farmland has a history of chemical fertilizers (NPK). Urea (N), calcium superphosphorus (P), and potassium sulfate (K) which has been used as inorganic fertilizer and applied at the rate of 150, 75, 75 kg/ha over the years to improve the fertility of the soil. The planting of Roma cultivar tomato seed was conducted in November 2020.

### Extraction of DNA and shotgun metagenomic sequencing

The DNA samples were extracted from the soil sample (5 g) collected with the aid of Nucleospin Soil Kit (Machery-Nagel, Germany) employing the direction as explained in the producer manual. After the DNA has been extracted, the quality of the extracted DNA was confirmed using Nanodrop. Quality DNA was sent to the Molecular Research Laboratory, United State of America for shotgun metagenomic assessment (Glass et al., 2010; Pereira et al., 2011). With the aid of the Nextera flex kit (Illumina, San Diego, CA, USA) the preparation of the library was conducted employing standard protocol. Evaluation of the concentration of the DNA was conducted with the aid of Life Technologies’ Qubit® dsDNA HS Assay Kit (Thermo Fisher Scientific, Waltham, MA, USA). With 50 ng of DNA, the library preparation was examined and its total concentration was quantified with the aid of Qubit® dsDNA HS Assay Kit (Waltham, MA, USA) and with the Agilent 2100 Bioanalyzer (Agilent, Santa Clara, CA, USA), the magnitude of the library was quantified. The libraries were combined and conducted with 0.6 nM ratios since their sizes vary in nature, and with 300 cycles employing Illumina NovaSeq 6000 system, pair-end sequencing was conducted.

### Downstream analysis and gene annotation

To an online web server MG-RAST (Metagenomics Rapid Annotation Subsystem Technology), the raw data were transferred (Meyer et al., 2008), and with the aid of SolexaQA to remove the low-quality reads, the sequences were subjected to quality filtering and dereplication of the metagenome data (Cox, Peterson & Biggs, 2010). Sequencing errors that occurred as a result of calibrating artificial duplicate reads (ADRs) were been removed with the aid of duplicate read inferred sequencing error estimation (DRISEE) (Callahan et al., 2019). The annotation of sequences follows after completing the assessment of quality control data against different datasets on the M5NR-database (Wilke et al., 2012) was analyzed employing the BLASTlike alignment (BLAT-algorithm) (Kent, 2002). With the aid of the SEED subsystem, rhizospheric microbial communities and various genes were classified following the method of Ye et al. (2005). The factors removed include 10^−5^ e-value and a lower limit to a subsystem with 60% sequence similarity. Other sequences that are almost 98% of the entire data not related to rhizosphere microbial communities sequences (archaea, fungi, and bacteria) were removed (Hong et al., 2019). To inhibit experimental error, default settings were used. Then, the percentage of the relative occurrence of putative plant growth functional metagenomes and disease-resistant genes was calculated after obtaining the mean of the sequence data for HR, DR, and BR employing MG-RAST. The sequences have the BioProject number PRJNA766489 on the NCBI SRA database.

### Statistical analysis

While conducting the annotation of sequences, rarefaction curves of the sequences were plotted on MG-RAST. The distribution and abundance of rhizosphere and bulk soil microbial communities at the phylum level and microbial phyla relative abundance plot was observed with the aid of a heatmap through z-score (Khomtchouk, Hennessy & Wahlestedt, 2017). *The* relative abundance of the plant-growth-promoting and disease-resistant genes was plotted with Circos software (Krzywinski et al., 2009). Diversity indexes and Pielou indices were employed to assess the diversity of the functional metagenomes in the soil samples and the comparison of the indices was conducted with the aid of the Kruskal-Wallis test. The PAST 3.20 version was used to analyze the data according to Hamel et al. (2018). To investigate the β diversity and assessment of the PGP and disease-resistant gene differences, PCoA and ANOSIM were employed (Carrell & Frank, 2015). With the aid of CANOCO version 5.0, the PCoA and PCA were plotted.

## Results

### Analysis of metagenome, quality control, and annotation of protein obtained

**Figure 1:**
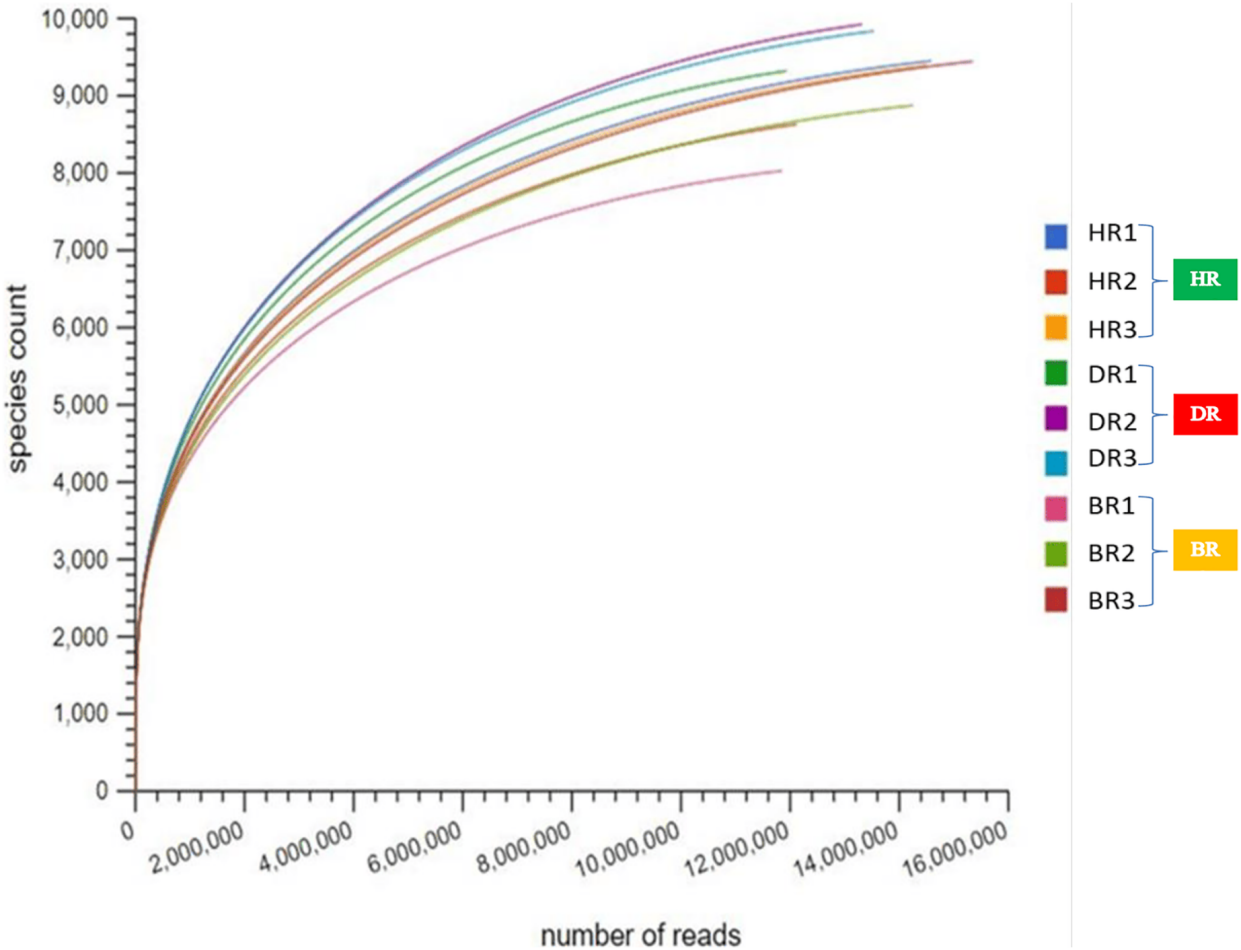
The rarefaction curve used to confirm species count across the tomato plantation sites. HR (1-3) = healthy tomato rhizosphere soil, DR (1-3) = diseased tomato rhizosphere soil, BR (1-3) = bulk soil.

After quality control (QC), the mean bp count of three replicates of the HR, DR and BR are 765,041,235, 1,352,124,415, and 1,385,218,693 respectively. The mean sequence counts obtained after QC of three replicates were 12,665,144, 11,966,280, and 5,247,459 for HR, DR, and BR (Table S1). After the sequences have been processed, the mean of predicted protein features of three replicates are HR (7,850,484), DR (4,208,874), and BR (11,448,097). The mean of identified protein after QC for three replicates of HR is 3,985,525, DR is 4,178,206 and BR is 2,515,439 (Table S1). However, the microbial species indices were revealed on the rarefaction curve obtained from MG-RAST as shown in Fig. 1.

### Microbial communities distribution in tomato plant rhizosphere soil

**Figure 2:**
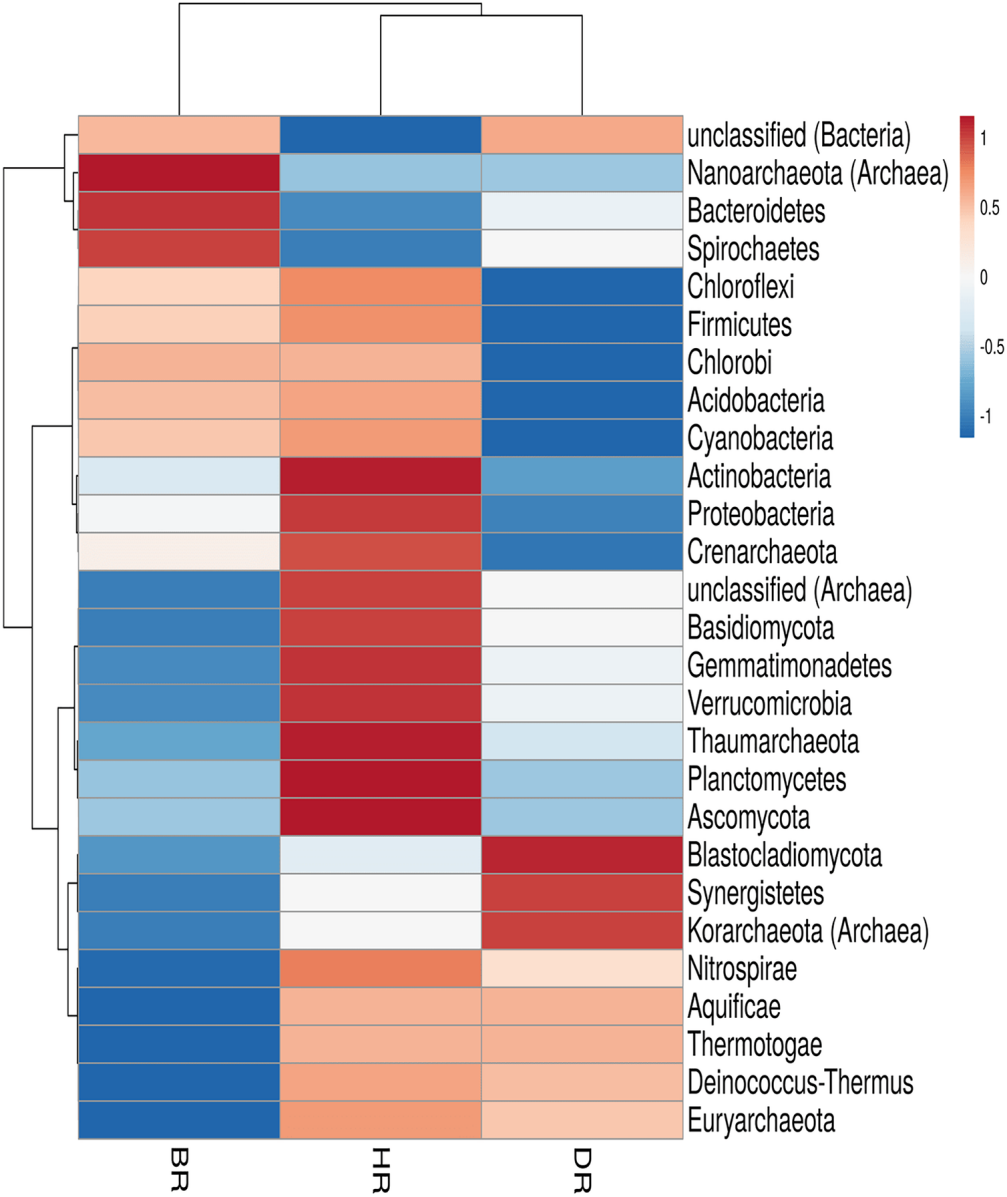
Heatmap of microbiomes phyla associated with the rhizosphere sample of the tomato plant and bulk soil. Each bar with distinct color revealed the saturation gradient with the mean value z-score. HR = healthy tomato rhizosphere soil, DR = diseased tomato rhizosphere soil, BR = bulk soil.

In the employed soil samples, the total number of microbes identified was twenty-seven (27). Eighteen (18) phyla were bacteria, six (6) phyla were archaea, and three (3) phyla were fungi. These phyla vary according to their dominance in the soil samples. There are nineteen (19) phyla present in the HR. The following listed were among the predominant bacteria dwelling in the HR; *Planctomycetes, Proteobacteria, Chloroflexi, Acidobacteria, Firmicutes, Cyanobacteria, Actinobacteria, Gemmatimonadetes, Verrucomicrobia, Deinococcus-Thermus, Nitrospirae, Chlorobi, Aquificiae*, and *Thermotoga*. There are three (3) prevalent archaea present in HR and they include; *Euryarchaeota, Thaumarchaeota*, and *Crenarchaeota*, and two (2) fungal phyla that are *Ascomycota* and *Basidiomycota* as observed in Fig. 2; Table S2. However, no significant difference was observed in the rhizosphere and bulk soil microbial communities phyla (Kruskal-Wallis, *p*-value = 0.89).

### Functional metagenomes in rhizosphere soil of tomato

There are fifty-two (52) functional genes obtained in both rhizospheres of tomato and bulk soil reported in this study. The obtained genes were categorized into PGP genes and disease-resistant genes.

#### PGP genes in tomato rhizosphere and bulk soil microbiomes

**Figure 3:**
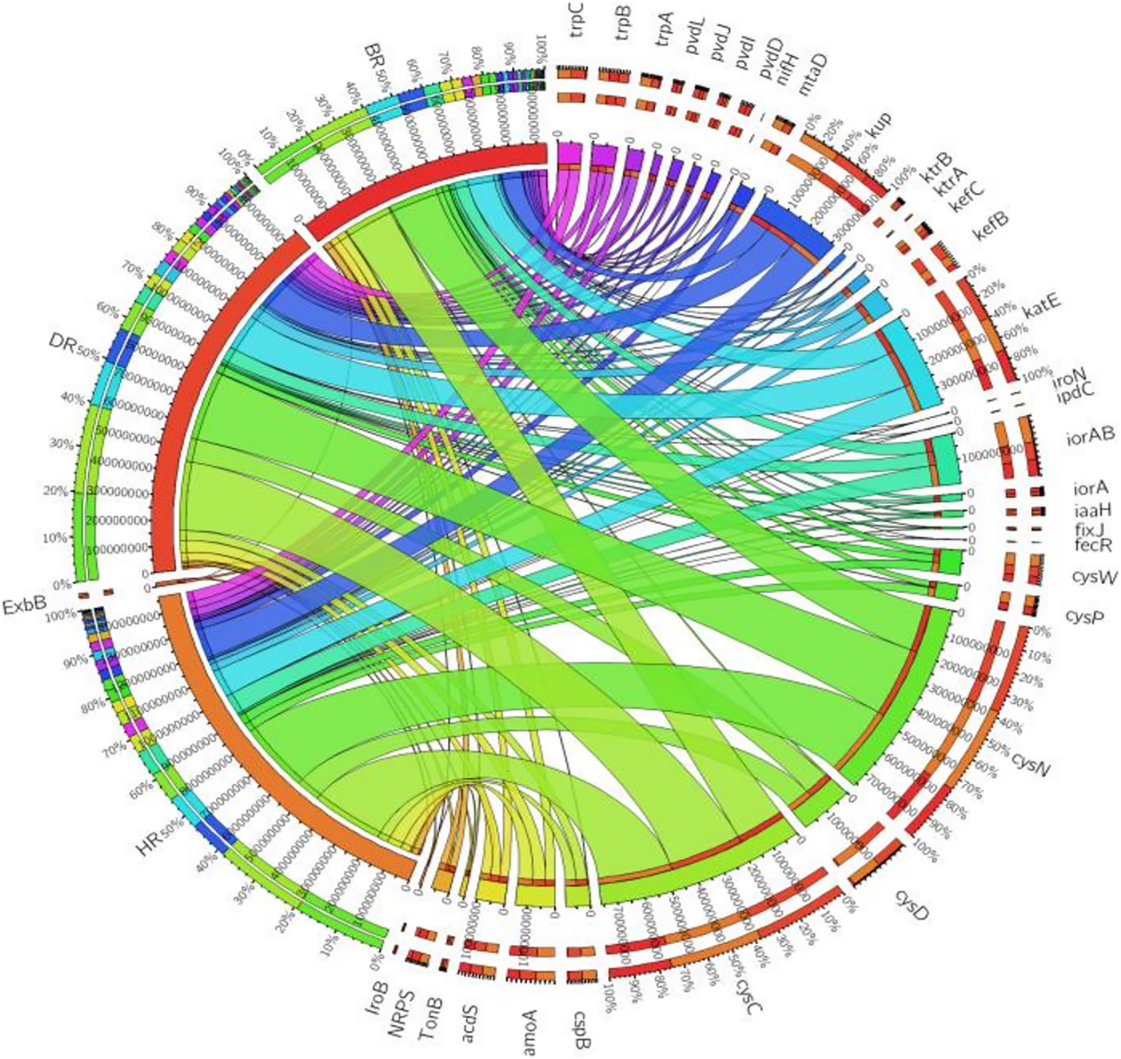
PGP genes relative abundance observed in the soil samples plotted to employ circos software. HR = healthy tomato rhizosphere soil, DR = diseased tomato rhizosphere soil, BR = bulk soil.

**Figure 4:**
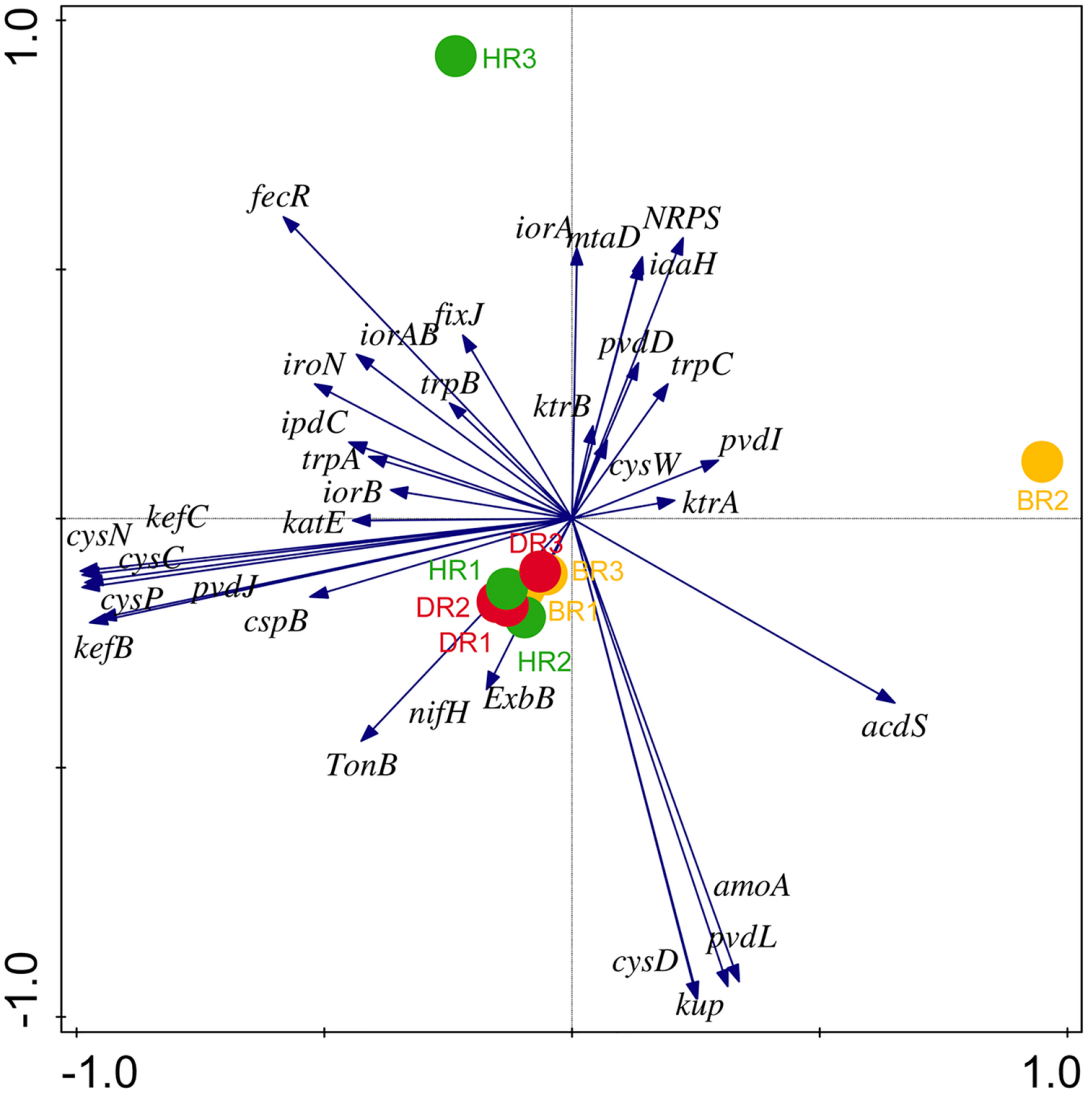
PCA graph of putative PGP metagenomes’ relative abundance. The vector lines symbolize the features of PGP metagenomes. A total of 44.0% on axis 1 and 24.8% on axis 2 reveal the variance based on the Bray-Curtis dissimilarity groupings. HR 1-3 = healthy tomato rhizosphere soil, DR 1-3 = diseased tomato rhizosphere soil, BR 1-3 = bulk soil.

There are thirty-four (34) putative PGP genes identified in the rhizosphere and bulk soil as shown in Fig. 3; Table S3 and they include; siderophore genes, ACC deaminase, nitrogen fixation genes, nitrification genes, IAA synthesis genes, sulfur metabolism genes, tryptophan biosynthesis gene, potassium cycling genes (Table 1). We also obtained the stress resistance gene (Fig. 3; Table S3). The following genes are abundant and greater in HR; siderophore genes (*iorB*, *NRPS*, *tonB*, *iroN, fecR, pvdD, pvdL*), ACC deaminase (*acdS, mtaD*), Nitrogen fixation genes (*nifH*, *fixJ*, *iorA, iorAB*), nitrification gene (*amoA*), IAA synthesis genes (*ipdC*), sulfur metabolism genes (*cspB, cysP, cysW*,), tryptophan biosynthesis gene (*trpA, trpB*, and *trpC*), and potassium cycling genes (*kef A, kefC, kup, ktrA*). The following genes were obtained in DR Siderophore genes (*ExbB, pvdJ*), Stress resistance gene (*KatE*), sulfur metabolism genes (*cysC, cysD*, and *cysN*), potassium cycling genes (*kef B* and *ktrB*) while in BR were found the siderophore gene (*pvdI*), and IAA synthesis genes (*iaaH*). From the abundance of the plant-promoting functional genes, there are no significant differences observed (*p* > 0.05). Figure 4 revealed the PCA graph of plant growth-promoting genes produced by microbial communities present in the rhizosphere soil was greater in the HR with the highest distribution.

**Table 1:** Plant-promoting growth and disease-resistant genes of tomato rhizosphere and bulk soil.

Type of genes	Form of genes	Genes present
Plant-promoting growth genes	Siderophore genes	*iorB*, *NRPS*, *tonB*, *iroN*, *ExbB, fecR, pvdD, pvdI, pvdJ, pvdL*
	ACC deaminase genes	*acdS, mtaD*
	Nitrogen fixation genes	*nifH*, *fixJ*, *iorA, iorAB*
	Nitrification gene	*amoA*
	IAA synthesis genes	*iaaH, ipdC*
	Sulfur metabolism genes	*cspB, cysC, cysD, cysP, cysW*, and *cysN*
	Tryptophan biosynthesis genes	*trpA, trpB*, and *trpC*
	Potassium cycling genes	*kefA, kefB, kefC, kup, ktrA*, and *ktrB*
	Stress resistance gene	*KatE*
Disease-resistant genes	Nucleotide-binding genes	*pstB*, *nadD*, *nadM, nrdD, nrdL, cpdB*
	Bacitracin transport system permease protein	*bceB, livH, dppB, dppC, pstA, pstC, ugpA, malG*
	Antimicrobial genes	*wcaG*
	Yersiniabactin non-ribosomal sequence genes	*Irp1, pchE, pchF*

#### Disease-resistant genes of the microbiomes in the employed soil samples

**Figure 5:**
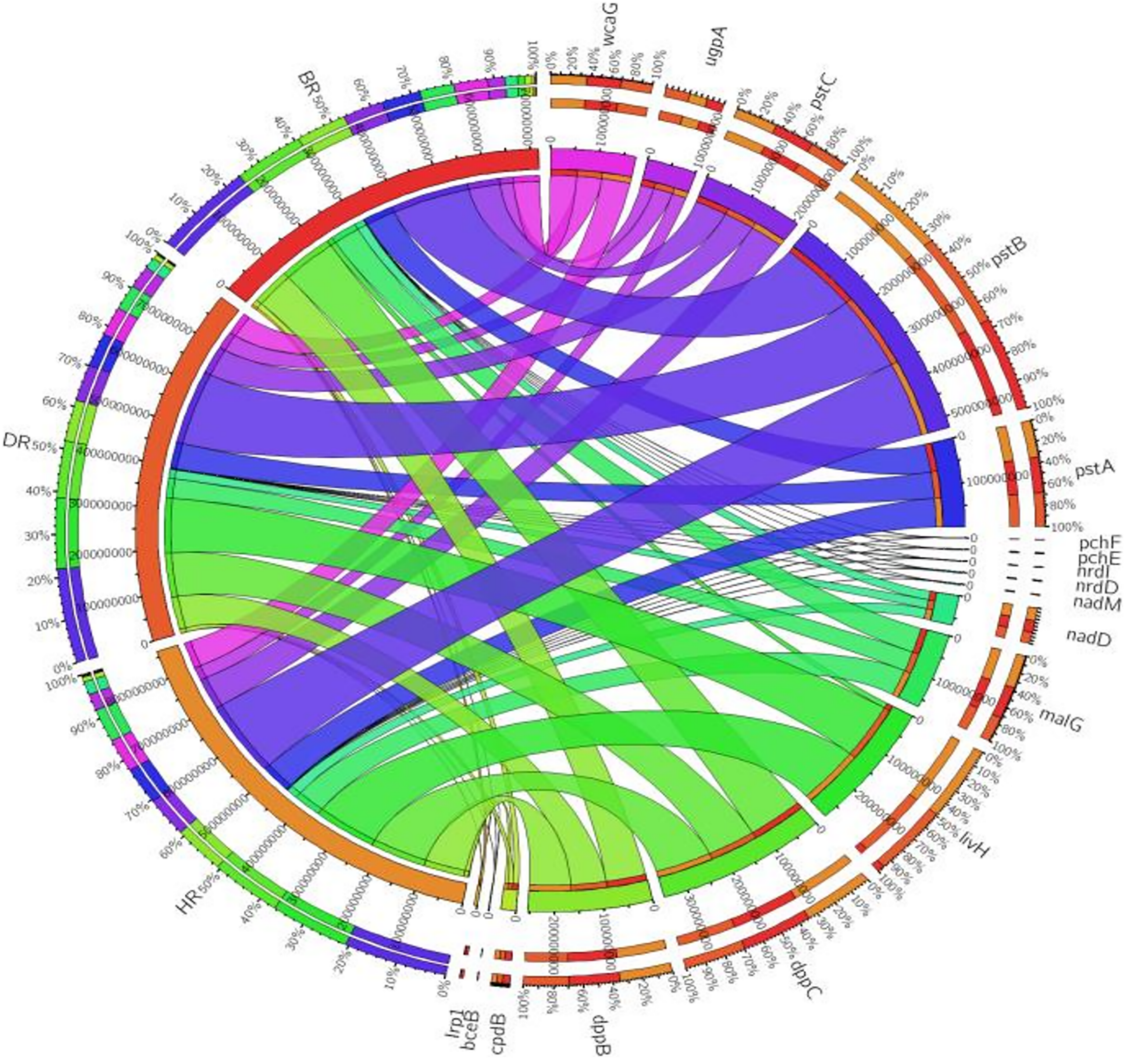
Disease-resistant genes relative abundance observed in the soil samples plotted to employ circos software. HR = healthy tomato rhizosphere soil, DR = diseased tomato rhizosphere soil, BR = bulk soil.

**Figure 6:**
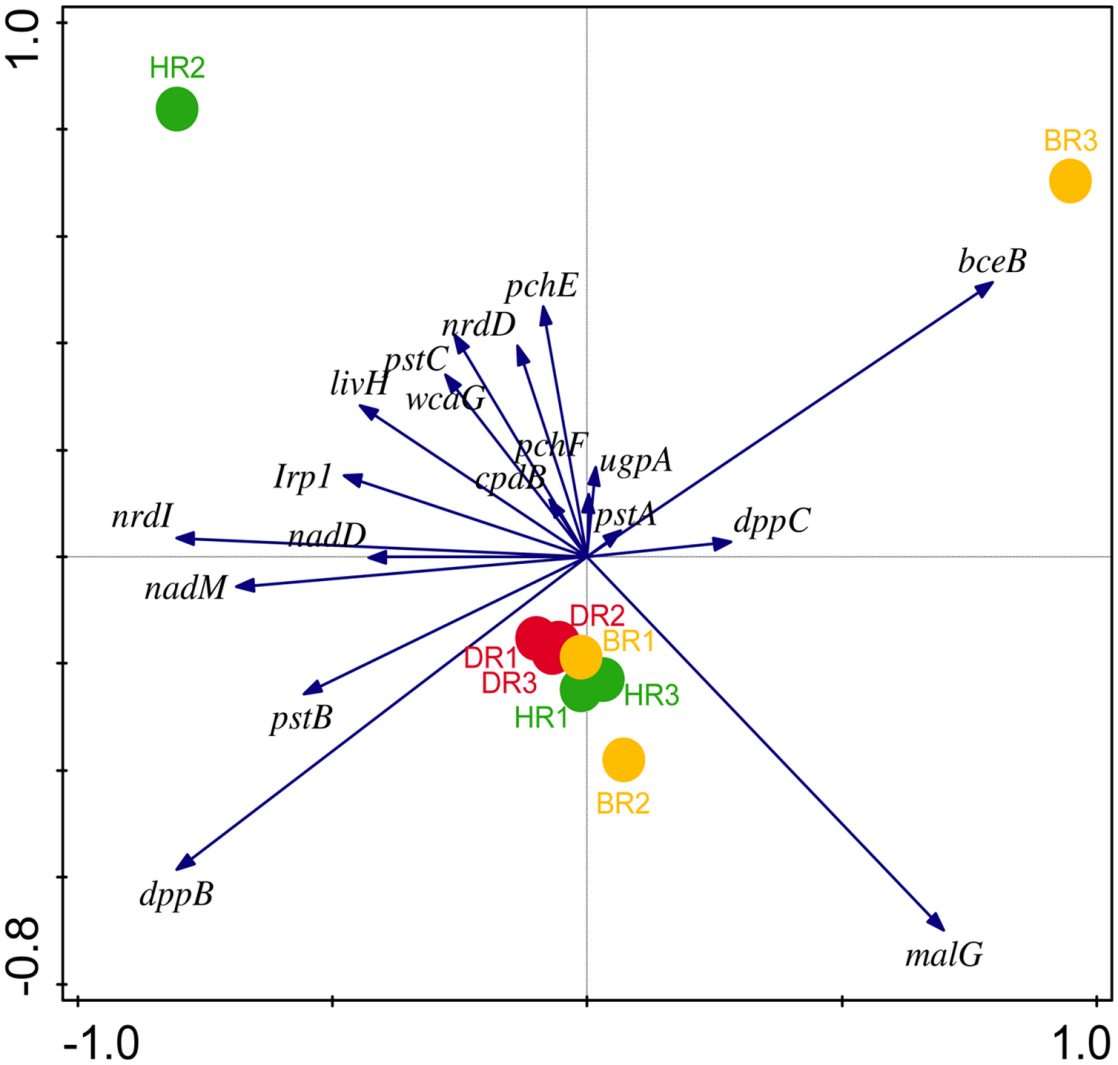
PCA graph of disease-resistant genes. The arrow symbolizes features of disease-resistant genes. A total of 46.6% on Axis 1 and 31.1% on-axis 2 reveal the variance based on the Bray-Curtis dissimilarity groupings. HR 1-3 = healthy tomato rhizosphere soil, DR 1-3 = diseased tomato rhizosphere soil, BR 1-3 = bulk soil.

Eighteen genes (18) were identified in disease-resistant genes. The following listed are some of the genes identified: nucleotide-binding genes, bacitracin transport system permease protein that helps provide energy for the transport of bacitracin across the cell membrane, and antimicrobial genes (Table 1). Others are yersiniabactin non-ribosomal sequence genes which include those found in Fig. 5; Table S4. Diseased-resistant genes observed in the rhizosphere and bulk soil in this study are; nucleotide-binding genes (*cpdB, pstB, nadD, nadM, nrdD, nrdL*), bacitracin transport system permease protein that helps provide energy for the transport of bacitracin across the cell membrane (*bceB, livH, dppB, dppC, pstA, pstC, ugpA, malG*). Others are antimicrobial genes (*wcaG*), and yersiniabactin non-ribosomal sequence genes which include (*Irp1, pchE, pchF*). In the abundance of disease-resistant genes, no significant difference was confirmed in the soil samples employed in this study. However, the distribution of disease-resistant genes identified in the rhizospheric organisms in the HR possessed the highest distribution as observed in Fig. 5; Table S4. The PCA plot revealed the distribution of PGP genes with 44.0% (Fig. 4) and disease-resistant genes with 46.6% variance (Fig. 6) in rhizosphere soil organisms inhabiting the HR. The vector’s arrows show the functional genes were influenced by the distribution.

### Alpha (α) and beta (β) diversity computation of plant growth functional microbial metagenomes in the soil

**Figure 7:**
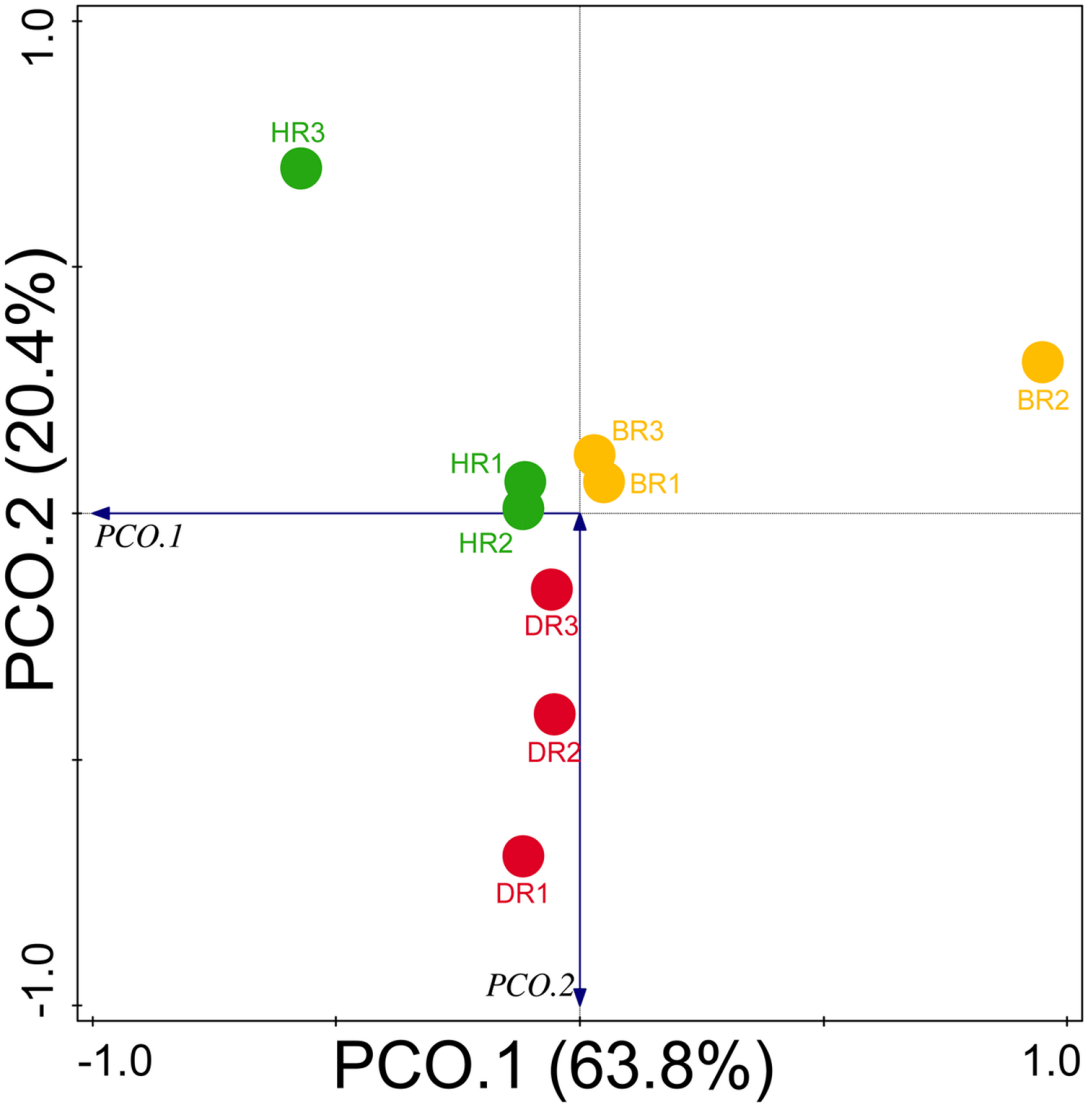
Principal coordinate analysis (PCoA) graph of PGP genes based on the Bray-Curtis dissimilarity grouping. HR 1-3 = healthy tomato rhizosphere soil, DR 1-3 = diseased tomato rhizosphere soil, BR 1-3 = bulk soil.

**Figure 8:**
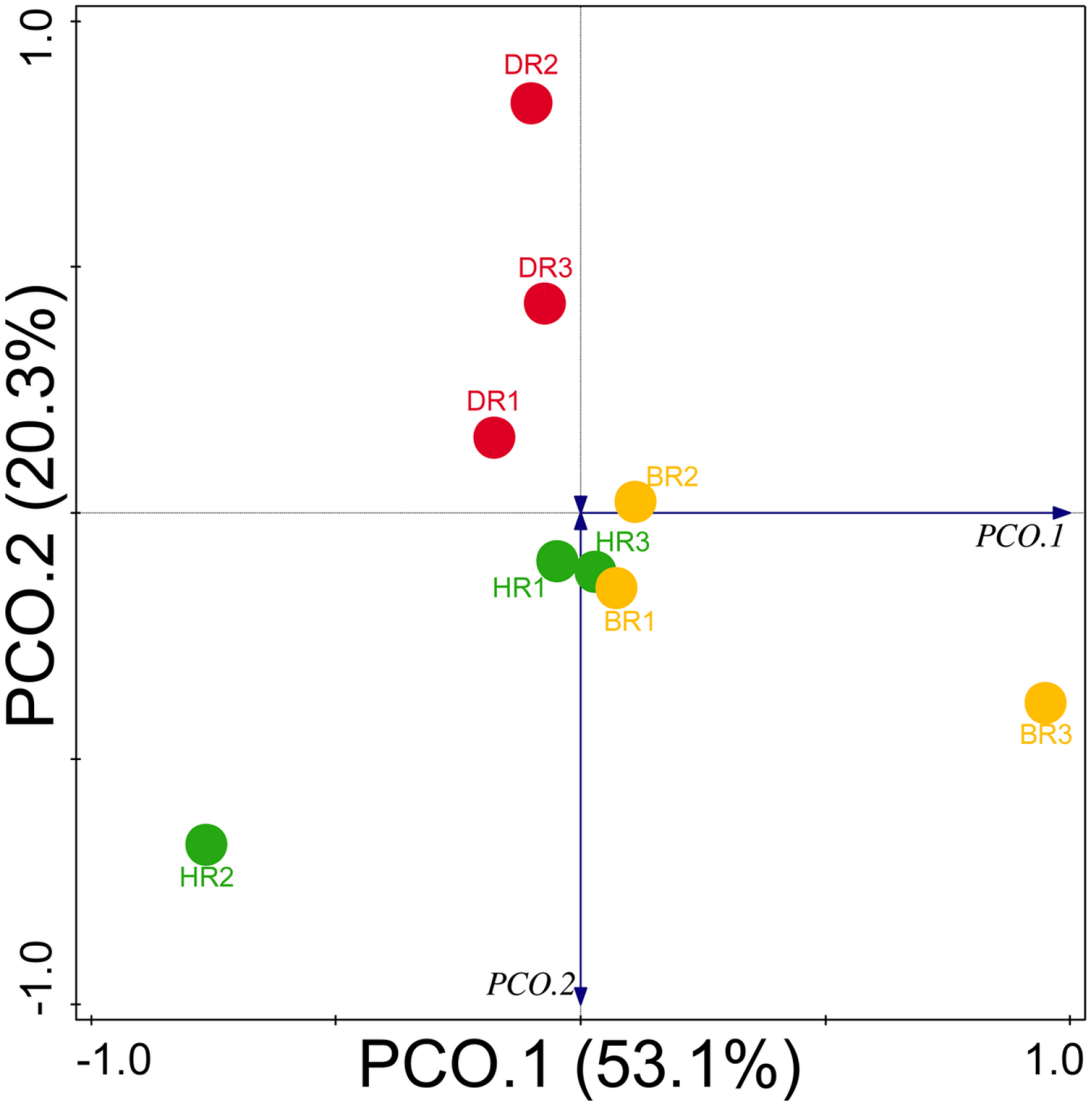
PCoA graph of disease-resistant genes based on the Bray-Curtis dissimilarity matrix. HR 1-3 = healthy tomato rhizosphere soil, DR 1-3 = diseased tomato rhizosphere soil, BR 1-3 = bulk soil.

Diversity indices and evenness were employed to calculate the α-diversity of the putative metagenomes in the soil samples. The diversity and evenness indices worked out the significant differences obtained in the genes of the soil samples analyzed as observed in Table 2 and the indices were not significantly different (*p* > 0.05). The results obtained for both plant-growth-promoting and disease-resistant genes showed no significantly different (Kruskal-Wallis, *p*-value = 0.72 and 0.94) in the soil samples (Table 2). However, a significant difference was confirmed for β-diversity employing analysis of similarity (ANOSIM) in the putative functional metagenomes of the soil samples obtained (*p*-value = 0.01; R = 0.67). Furthermore, PCoA unveiled a distinct separation in the genes as observed in Figs. 7 and 8. This Euclidean dissimilarity matrix-based plot revealed the distribution of the functional genes in microbial communities in HR compared to DR and BR.

**Table 2:** The diversity indices of PGP genes and disease-resistant genes in microbial communities of rhizosphere and bulk soil of tomato plant.

Diversity indices	HR	DR	BR	*p*-value
PGP genes				
Simpson	0.91 ± 0.001	0.90 ± 0.0003	0.91 ± 0.0004	0.76
Shanon	2.80 ± 0.01	2.73 ± 0.05	2.84 ± 0.005	
Evenness	0.48 ± 0.03	0.45 ± 0.003	0.50 ± 0.002	
Disease-resistant genes				
Simpson	0.88 ± 0.05	0.88 ± 0.02	0.88 ± 0.003	0.93
Shannon	2.32 ± 0.03	2.31 ± 0.003	2.31 ± 0.02	
Evenness	0.48 ± 0.04	0.48 ± 0.02	0.48 ± 0.05	

**Note:**

*p*-values obtained from the Kruskal-Wallis test. HR, healthy tomato rhizosphere soil; DR, diseased tomato rhizosphere soil; BR, bulk soil.

## Discussion

The shotgun metagenomic sequencing was conducted on healthy, diseased tomato plant rhizosphere as well as bulk soil in this research. The analysis of the raw data was performed on the web service MG-RAST to reveal the microbiomes dwelling in the soil samples of tomato rhizosphere and bulk soil. Some of the mentioned microbial communities has been reported as renowned tomato plant growth-promoting microorganisms (Adedayo, Fadiji & Babalola, 2022a) and other studies have reported the potential of the microorganisms to produce significant genes that contribute to plant growth and improvement of plant health status (Buée et al., 2009; Orozco-Mosqueda et al., 2019). Recent studies had explained how rhizosphere microorganisms improve the growth of tomato plants through direct and indirect mechanisms (Fadiji & Babalola, 2020; Richardson et al., 2009). Direct mechanisms involved 1-aminocyclopropane-1carboxylate (ACC) utilization, phosphate solubilization, phytohormones production, and siderophore production (Ajilogba & Babalola, 2013; Babalola, 2010) while the indirect mechanism involved biological control potentiality, secondary metabolites production, hyper-parasitism, plant resistance (Ajilogba, Babalola & Ahmad, 2013; Oloyede et al., 2021).

Siderophore genes contribute to the development of tomato growth by producing various genes as reported by Etminani, Yousefvand & Harighi (2020), NRPS gene encodes siderophore biosynthesis non-ribosomal peptide synthetase modules are abundant in HR. These genes incorporate amino acids in the growing chain, thereby impacting the structural diversity of tomato plants. Our study corresponded with the study of Collemare et al. (2014) which revealed how the gene is involved in a series of reactions that involve amino acid activation, binding, selection, modification, peptide elongation, and release. *tonB* gene that encodes ferric siderophore transport system, periplasmic binding protein is produced by *Pseudomonas fluorescens* Pf-5 that perform the function of assimilation of siderophores, high-affinity iron-chelating compounds, and other nutrients from the environment and greater in HR. The genes attached to high affinity to particular substrates outside the environment of the cell at the initial stage of the energy-dependent transport of the substrate into the periplasmic space (Hartney et al., 2011). *iroN genes* encode Iron protein was abundant in HR. Our study is in line with Etminani, Yousefvand & Harighi (2020) who reported how *iroN* siderophore genes improve the development of tomato plantations by contributing to the growth and ripening of the tomato fruits. *ExbB* genes encode ferric siderophore transport system, and biopolymer transport protein. The genes were greater in DR according to our study. They perform the roles with *TonB* genes to transport the energy of the proton-motive force to outer membrane receptors in *Serratia marcescens* for the assimilation of iron. Schätzle et al. (2021) also reported how these genes were produced by *Pseudomonas putida* WCS358 and involved in ferric-pseudobactin transport and siderophore production. *fecR* and *pvdD* genes that encode iron (III) dicitrate transmembrane sensor protein and pyoverdine sidechain non-ribosomal peptide synthetase were abundant in the HR. Due to their abundance in HR, our report coincides with Roca et al. (2013) that revealed how these genes were produced by *Pseudomonas putida, *and possessed the potential to induce pyoverdine thereby promoting phosphorous and iron solubilization and stimulate phytohormones under iron-deficient conditions to promote the healthy living of the plant. *pvdI*, and *pvdJ* gene that encodes pyoverdine sidechain non-ribosomal peptide synthetase, pyoverdine sidechain non-ribosomal peptide synthetase respectively. *pvdI* gene is abundant in BR while *pvdJ* gene is abundant in DR. *pvdI, pvdJ* and *pvdD* genes were peptides chain genes located on the outer membrane pyoverdine receptor (*fpvA*) of fluorescent *Pseudomonas* spp. that are divergent genes in the region, they reveal the coevolution that keeps the specificity of the siderophore for the receptor (Gross & Loper, 2009). Visca, Imperi & Lamont (2007) also reported how PVD synthesis genes *pvdL, pvdI, pvdJ*, and *pvdD* obtained from *P. aeruginosa* strain PAO1 comprise various enzymes which partake in the synthesis of secondary metabolites by microbes. These genes code for non-ribosomal peptide synthetases and perform specific functions in the biological synthesis of pyoverdine by *P. putida*. Pyoverdines are category of diffusible and fluorescent siderophores that act as the primary iron asimilation of the *Pseudomonas* species thereby preventing angular leaf spot disease of strawberries (Henry et al., 2016). The enzymes capacitate the formation of the peptide bond between amino acids that cannot be integrated by ribosomal synthesis, and this is relevant to amino acid residues found in PVD obtained from *P. aeruginosa* PAO1.

ACC deaminase genes are involved in the production of plant-produced aminocyclopropane-1-carboxylic acid (ACC) that is produced as a result of the plant stress hormone ethylene reducing the effect of the plant to diseases. *acdS* and *mtaD* that encode 1-aminocyclopropane-1-carboxylate deaminase and methylthioadenosine deaminase respectively were ACC deaminase genes identified in this study and were greater in HR. Kang et al. (2019) revealed the potential of *acdS* gene to improve the growth of tomatoes and further tolerate salinity stress as a result of the regulation of secondary metabolite which goes with our study for being abundant in HR. *mtaD* gene induces plant growth, improves fruit ripening, and produce stimulates the production of the enzyme that belongs to the family of metallodependent hydrolases and involves phosphonates metabolism, together with various pesticides and herbicides (Onishchuk et al., 2015). This gene is known for preventing the poisoning of crop plants caused by chemical pesticides and herbicides applied to farmland (Loeschcke & Thies, 2015; Onishchuk et al., 2015).

Nitrogen fixation (nif) genes code for proteins associated with and related to the fixation of atmospheric nitrogen into a form of available nitrogen to plants. We also find microbiomes that produce *nifH* and *FixJ* that encode nitrogenase (molybdenum-iron) reductase and maturation protein and two-component nitrogen fixation transcriptional regulator synthesize the nitrogen fixation were highly abundant in the HR site. Other genes found abundant for the metabolism of nitrogen fixation include *iorAB* and *iorA* encode indolepyruvate ferredoxin oxidoreductase, alpha and beta subunits, and indolepyruvate oxidoreductase subunit (EC 1.2.7.8). They were all abundant in HR contributing to the growth and health status of the tomato plant in this study. Imada et al. (2017) reported how *iorA* is upregulated by tryptophan, showing the main function of these genes in IAA biosynthesis. Our result relates to the result presented by Guerrieri et al. (2020) which explained how microorganisms fix nitrogen (N) in the rhizosphere soil of tomatoes, thereby improving its growth. N is a macroelement/nutrient required in abundance for the growth of plants. This element plays an important role within the plant to make available energy when and or where the plant needs it to optimize its production.

Masood, Zhao & Shen (2020) reported the plant growth-promoting bacteria (PGPB) *Bacillus pumilus* that can fix atmospheric N_2_ to the *Solanum lycopersicum* rhizosphere soil with the aid of *nifH* gene produced by the PGPB and therefore used to simplify the application of N fertilizer in agricultural practice. The uptake of N to the tomato plant modifies the development of the tomato and improves soil NH_4_^+^ concentration. N also contributed to the increase in biomass of the rhizobacteria in the rhizosphere soil, increases expression of *nifH* gene, and the activity of soil nitrogenase when the soil is inoculated with *B. pumilus* (Masood, Zhao & Shen, 2020). *amoA* gene encoded ammonia monooxygenase structural gene is abundant in HR. The gene involved in nitrification of the microbial process whereby nitrogen compounds were reduced from ammonia and oxidized to nitrite and nitrate (Singh, Choudhary & Sharma, 2020).

IAA (indole acetic acid) genes contribute to the production of important hormones like Gibberellins (GA) and IAA hormones. The biological synthesis of the auxin indole-1-acetic acid (IAA) from tryptophan is being encoded Indoleacetamide hydrolase (EC 3.5.1) *iaaH* gene abundant in BR in this study. This gene can express itself in the seed of tomato fruit thereby conferring treatment on the fruit with the naphthalene acetamide (NAM) regarded as an auxin precursor, that is hydrolyzed to naphthalene-1-acetic acid (NAA) by indoleacetamide hydrolase (*iaaH*-encoded enzyme) (Carmi et al., 2003). *ipdC* (indole-3-pyruvate decarboxylase encoding) gene is an important gene in the overall IAA biosynthesis (Steenhoudt & Vanderleyden, 2000). This gene is abundant in HR according to our study and involved in a specific function in the production of auxin hormone which aids the growth of plants at the shoot and the elongation of plant roots. Our result is consistent with the report of Khan et al. (2014) that explained how GA and IAA hormones are produced in *Spinghomonas sp*. LK11 improved the growth of the tomato plant. GA is an important plant hormone controlling major mechanisms in plants. This hormone is of substantial value in agricultural processes that include flowering, fruit patterning root and shoot elongation, and seed germination. Various studies have reported how important GA movement is for multiple developmental processes, how GAs induce elongation of filaments by increased elongation of cells, and improve the opening of anther to the safe passage of pollen grains to the stigma (Hartweck, 2008; Marciniak & Przedniczek, 2019; Wheeldon & Bennett, 2021). IAA is also an important hormone in the plant that activates differentiation, cell division, and other extensions in plants. The genes activate the growth and development of seed and tuber crops in plants, improving the vascular tissues (phloem and xylem) embedded in the root, promoting lateral initiation, taking control of the activity of vegetative propagation, and elongation of the root (Wei et al., 2019). This hormone also assists in the biological synthesis of metabolites, photosynthesis, production of pigments and light and fluorescence, controlling gravitational responses, and resistance to extreme conditions (Hassan, 2017). Our result reveals that at HR sites, the genes obtained in rhizosphere soil employed in this study contributed to the significant growth of tomato plants. This organic catalyst inhibits the synthesis of ethylene production in plants and assists in the hydrolysis of ACC. Another hormone that promotes the germination of the seed is ethylene, it controls the colonization or inhabitation of bacteria in the plant tissues (Babalola, 2010). Over-production of this hormone can destroy the growth and health status of a plant (Djanaguiraman, Prasad & Al-Khatib, 2011; Jiroutova, Oklestkova & Strnad, 2018). Some studies also reported that ammonia and α-ketobutyrate were produced as a result of the hydrolysis of ACC by ACC deaminase thereby making available essential nitrogen that promotes the growth of microorganisms (Ali & Kim, 2018; Danish & Zafar-ul-Hye, 2019). Microbial communities like *Actinomycetes* particularly *Streptomyces filipinensis* inhabiting the rhizosphere soil of the tomato plant were reported by El-Tarabily (2008) to produce ACC deaminase as well as IAA and further contributed to the growth and development of the tomato plant.

Sulfur metabolism genes carry out important various functions that include the following; balance redox reaction, cell differentiation, cell death, defense against biotic and abiotic stresses, detoxification of xenobiotics, protein folding, and phytochelatin precursors. *cspB* gene encoding cold shock protein is highly present in the HR site where they confer protection against environmental stress on tomato plants. The gene controls plant RNA and assists plant cells to manufacture proteins that are necessary for growth and aid the production of a bountiful harvest in a drought environment. Paul, Nuccio & Basu (2018) support our study which revealed the potential of *cspB* gene produced by certain microorganisms in the HR. Psychrophilic *Bacillus* spp. reveals how *cysC*, and *cysD* genes encoded adenylyl-sulfate kinase and sulfate adenylyltransferase subunit 2 respectively were abundant in DR. These genes also protect tomatoes from environmental stresses that can cause disease to the tomato plant. Our result concurs with the study of Zubair et al. (2019) which explained how wheat plants were been protected from disease invasion and tolerate cold stress. The genes *CysP-*encoded sulfate and thiosulfate binding protein were greater in the HR site while *CysW-*encoded sulfate transport system permease protein and *CysN* gene-encoded sulfate adenylyltransferase subunit 1 were greater in the DR site. Kang et al. (2020) revealed how *CysP* is the sulfate transport protein, controlled by sulfate: H^+^ symport. However, the role of the gene was justified by incorporating *B. subtilis* producing *CysP* in *E. coli* with a mutated sulfate transport system. This encoded gene in *B. subtilis* made up of genes partaking in sulfur metabolism procedures, like the sulfate adenylyltransferase gene. Cheng et al. (2017) reported how *Pseudomonas fluorescens* produced *cysH* adenylsulfate reductase gene that carries out the role of induced systemic resistance against plant diseases, biosynthesis of auxin, biosynthesis of steroids, carbohydrate metabolism, sulfur uptake, and transport and promote Arabidopsis plant growth.

Tryptophan biosynthesis genes (*trpA* gene-encoded tryptophan synthase alpha chain (EC 4.2.1.20), *trpB* gene-encoded tryptophan synthase beta chain (EC 4.2.1.20), and *trpC* gene-encoded tryptophan biosynthesis protein TrpCF) were among the reported genes in this study contributing to the well-being of the tomato plant with *trpA* and *trpC* abundant in HR site and *trpB* in the BR site. These genes encode the biological synthesis of enzymes for the amino acid tryptophan. Asaf et al. (2018) explained how the gene cluster of biosynthesis of tryptophan (*trpA, trpB*, and *trpD*) can produce IAA biosynthesis in *Sphingomonas* sp. LK11 during genome sequencing of endophytic *Sphingomonas* sp. LK11 and its ability to improve soybean plant growth.

Potassium cycling (kef) genes reduce the internal pH and protect the plant from the acidic nature of the soil. *kefC* encoding glutathione-regulated potassium-efflux system protein KefC was abundant in the HR site while *kefB* gene encoding glutathione-regulated potassium-efflux system protein KefB is abundant in DR. These genes control the potassium cycling system in other to improve the growth of tomato. The full stimulation of *KefC* requires *KefF* in other to be regulated by glutathione (GSH) and its S conjugates (Lyngberg et al., 2011). Hu et al. (2021) study was in line with our report on *kefB* gene in DR, the gene was upregulated in the antibacterial mechanism of Biochanin A to control *Xanthomonas axonopodis* glycines a pathogen that causes bacterial pustule disease in soybean. *Kup* gene-encoded system potassium uptake protein is greater in BR. This performs a special function by maintaining osmotic balance in the response of plants to abiotic stress by transporting potassium ions (Ou et al., 2018). *Kup* gene was reported to be produced by *Microbacteriaceae* (GZ3), *Frankiaceae* (GZ3) inhabiting the grassland thereby promoting potassium uptake according to Chukwuneme, Ayansina & Babalola (2021). The potassium uptake protein, integral membrane component (*ktrA*) gene is abundant in HR while potassium uptake protein integral membrane component (*ktrB*) genes were abundant in DR according to our study. These genes produced by microbial communities living in the rhizosphere of the growing plant solubilize the insoluble K to soluble forms for plant assimilation, aid plant growth, and enhance the abundant harvest of the crop. *ktrA* gene was reported by Samaras et al. (2021) to be produced by *Bacillus subtilis* to support and improve the growth of cucumber thereby maintaining the health status of the plant. *B. subtilis* isolated from *Vitis vinifera* rhizosphere soil produce *KtrB* genes that encourage the survival of the plant in high salinity environments (Leal et al., 2021).

Stress-resistant genes *katE* and *cspB* encoded catalase HPII and cold shock protein were not left out of the genes detected in this study. *katE* gene is abundant in DR while *cspB* gene is abundant in HR. The genes function by protecting bacterial cells through the function of a knocked-out gene that is associated with response to the stress toward the organism. Our result is similar to the study conducted by Taheri & Tarighi (2012) that showed how stress-resistance genes producing peroxidase, chitinase, and lignin formation demonstrated basal resistance to fungal phytopathogen *Rhizoctonia solani* of tomato. *KatE* gene helps to detoxify endogenous or xenobiotic compounds and also contributes to oxidative stress metabolism in plants. It contributes to the improvement of resistance against various phytopathogens like *Fusarium oxysporum*, and *Escherichia coli* on tomato fruits (Moghaieb et al., 2021). *CspB* genes were abundant in HR in this study where they prevent various stresses on the tomato plant. Our findings acknowledged the report that revealed how this gene is produced by *Bacillus subtilis* to promote tolerance to abiotic stress, maintaining growth and photosynthesis in various plants like rice, maize, and Arabidopsis (Reguera, Peleg & Blumwald, 2012). The organisms producing these genes were reported to show resistance against *Oidium neolycopersici* in tomatoes thereby decreasing the hypersensitive response and sporulation of *O. neolycopersici* in tomatoes (Pei et al., 2011).

Various microbial communities have also been reported as tomato plant disease-resistant microorganisms (Choi et al., 2020; Roy et al., 2019). The bacteria species which include *Acidobacteria, Gemmatimonadetes, Chloroflexi*, and *Actinobacteria*, and the fungi *Glomeromycota, Kribbella, Niastella, and Preussia* otherwise have the potential to produce important genes that act against various diseases thereby controlling the phytopathogen invasion biologically without the introduction of chemical derivatives for improvement of tomato plant health status (Samaddar et al., 2021). In this study, the disease-resistant genes take part in various pathways that involve the production of nucleotide-binding genes, bacitracin transport system permease protein, isopenicillin N epimerase genes, yersiniabactin non-ribosomal sequences genes, resistance to *L. maculans* genes, and Leptosphaeria resistance genes.

These genes are produced by microbial communities inhabiting the rhizosphere soil of tomatoes to prevent diseases produced by spoilage organisms. *Bacillus amyloliquefaciens* produced antibacterial compounds that biologically control bacterial wilt of tomato pathogens *Ralstonia solanacearum* (Raza et al., 2016). These bacteria produced various genes that code for the synthesis of antibacterial compounds and inhibit the invasion of bacterial wilt of tomatoes. A huge number of *Bacillus* gene clusters were identified in the healthy rhizosphere of tomato plants. Biosynthetic gene clusters (BGCs) gene code for the synthesis of the following compounds; bacteriocins (bacillibactin, fengycin, subtilin, surfactin), *PKs*, *NRPs*, *PKs-NRPs*, terpenes, *etc*., that have antimicrobial activity against *Botrytis cinerea, Erwinia carotovora*, *Pseudomonas syringae, Phytophthora infestans, Rhizoctonia solani*, and *Verticillium dahlia* (Zhou et al., 2021).

The nucleotide-binding genes protect the DNA of the microorganisms in the rhizosphere of tomato after the DNA has been degraded by DNase and RNase present in the extracellular fluid and promote its establishment into innate immune cells. *cpdB* gene-encoded 2′,3′-cyclic-nucleotide 2′-phosphodiesterase was abundant in DR. The gene was believed to be abundant in DR following the report on how it is been produced by PGPR stains (*Pseudomonas* sp. and *Bacillus* sp.) that perform the role of biocontrol PGPB, plant growth promotion, and plant stress homeo-regulation (Suresh & Abraham, 2019). The gene was known for controlling phytopathogens in the tomato plant in this study. Our research coincides with the report of Liu & Filiatrault (2020) that reveal how the gene promotes the antibacterial activity of potassium tetraborate tetrahydrate (PTB) on *Pectobacterium atrosepticum* SCRI1043 wild-type that causes soft-rot bacterial phytopathogens. Nicotinate-nucleotide adenylyltransferase (EC 2.7.7.18) (*nadD*) gene is abundant in HR produced by microbial communities inhabiting the rhizosphere and carries out the function of defense against phytopathogens. Our study is in line with Pétriacq et al. (2012) who acknowledged how *nadD* gene is involved in signaling pathways that are associated with defense responses. Phosphate transport ATP-binding protein (TC 3.A.1.7.1) *pstB* gene is a nucleotide-binding gene that performs the role of phosphate uptake in HR. According to Asaf et al. (2018), the gene was reported for removing inorganic phosphate transport in *Bacillus subtilis* and *Escherichia coli* to improve their resistance potential and promote plant growth which corresponds to our study for improving tomato plant growth. *nrdD* gene-encoded anaerobic ribonucleoside-triphosphate is predominant in the BR site. It functions by converting ribonucleotides into deoxy-ribonucleotide that synthesize and repair DNA. Buttimer et al. (2020) reported how *nrdD* and *nrdG* nucleotide genes produced by the bacteriophage *Certrevirus* involved in DNA methylation and nucleotide metabolism thereby conferring resistance to potato blackleg and soft rot diseases caused by phytopathogen *Pectobacterium atrosepticum*.

The bacitracin transport system permease proteins (*bceB*) gene is prevalent in the HR site. They revealed tremendous action they carry out against phytopathogens thereby inhibiting the proliferation of diseases. Enebe & Babalola (2019) unveiled how the *bceB* (bacitracin transport system permease protein) gene potential to suppress the growth of the disease in maize plants. For a mutual association in bacterioid structures of *Rhizobium leguminusarium*, branched-chain amino acids are important hydrophobic and hydrophilic components of the bacterial membrane. *livH* genes encoded high-affinity branched-chain amino acid transport system permease protein (TC 3.A.1.4.1) is abundant in HR. The gene is associated with periplasmic binding proteins (*livJ* or *livK*) and improves the assimilation of branched-chain amino acids. *LivH* genes are found in the inner membrane encoding the hydrophobic and hydrophilic proteins (Zuniga-Soto et al., 2019). *dppB* and *dppC* genes encoded dipeptide transport system permease protein (TC 3.A.1.5.2) and dipeptide transport system permease protein (TC 3.A.1.5.2) were prevalent in HR and they were involved in dipeptide transport across bacteria membrane. According to Carter et al. (2002), the *dppABCDF* genes found in *R. leguminosarum* correspond to that in other bacteria species. The distance between *dppA* and *dppB* contains inverted repeats that may modify mRNA and genes involved in the translocation of the substrate across the membrane of *Escherichia coli*. *pstA, pstB* and *pstC* genes encoded phosphate transport system permease protein (TC 3.A.1.7.1), phosphate transport ATP-binding protein (TC 3.A.1.7.1), and phosphate transport system permease protein (TC 3.A.1.7.1) were abundant in HR site. The genes were responsible for the protein binding system for phosphate transportation and other substrates across the membrane of the bacteria required to support the growth of the tomato plant. Our report is consistent with the report of Kang et al. (2020), pst operon genes made up of *pstS, pstC, pstA, and pstB* genes together with *phoP* or *phoR* involved in a two-component signal transduction system mainly for assimilation of phosphate in *B. subtilis* and *E. coli*. *UgpA* gene-encoded glycerol-3-phosphate ABC transporter, permease protein (TC 3.A.1.1.3) involved in binding protein-dependent transport system for sn-glycerol-3-phosphate, transport substrates through the membrane, and abundant in DR site. UDP-glucose synthetic pathway was reconstructed in *E. coli* by inserting the cellobiose *ugpA* gene obtained from *Bifidobacterium bifidum* for controlling acetic acid accumulation in the bacteria (Pei et al., 2019). *malG* gene-encoded maltose/maltodextrin ABC transporter 2, permease protein carries out the function of transporting maltose through the inner membrane of the bacterial cell and further catalyzes the maltose hydrolysis to glucose. This gene is abundant in the HR site according to this study. Mächtel et al. (2019) likewise reported the transport mechanism of the maltose ABC importer across biological membranes. The aggregation of biochemical, biophysical, and structural studies has produced the maltose transporter as *MalF* and *MalG* as the transmembrane domains of nucleotide-binding domains.

Nucleoside-diphosphate-sugar epimerases (*wcaG*) gene is known for reducing the virulence activity of phytopathogens in the plant. This gene is highly abundant in the HR site in this study. our study coincides with the nature of antimicrobial genes (*wcaG*) regarded as inactivated genes that possessed the potential to reduce the virulence activity of a gram-negative bacterium *Pectobacterium carotovorum* that initiates soft rot disease on fruits, vegetables, and other crop plants through the exoproteins activity that contains plant cell wall lysis enzymes (PCWDEs). This gene encodes NAD-dependent epimerase which is a homologue of GDP-fucose synthetase of *Escherichia coli*. GDP-fucose synthetase takes part in the biological synthesis of colanic acid (CA), an exopolysaccharide in *E. coli*. The *wcaG* mutants of *P. carotovorum* initiated an improved stage of biofilm compared to their parent (Islam et al., 2019).

Other genes reported in this study include yersiniabactin non-ribosomal sequence genes. They also have the potential to promote disease resistance in plant crops. Iron acquisition yersiniabactin synthesis enzyme polyketide synthetase (*Irp1*) gene performs a specific function in the acquisition of iron *in vitro* and *in vivo* and is abundant in the HR site. This gene was known for its action of resisting phytopathogens on tomato plants in this study. According to Liu et al. (2021), *irp1* polyketide synthase gene is also regarded as the yersiniabactin YBT gene cluster of *Yersinia pestis* produced by *Pseudomonas syringae. pchE* genes encoded dihydroaeruginoate synthetase, non-ribosomal peptide synthetase modules abundant in HR and *pchF* encoded pyochelin synthetase, non-ribosomal peptide synthetase module are abundant in BR site. The genes produced by microbial communities inhabiting soil carry out various functions among which are pyochelin biosynthesis, uptake, and regulation (Rampioni et al., 2010), recruit iron, detoxification metal, or ROS (reactive oxygen species) generation. The biological synthesis of pyochelin begins with the BGC composing *pch*DHIEFKCBA operon. *pchA* and *pchB* initiate the synthesis and the isochorismate is transformed into salicylate by *pchB*. The salicylate produced is initiated by *pchD* while *pchC* eradicates error-charged molecules. Then *pchE* and *pchF* add up L-cysteine residues and carry out cyclization and epimerization, thereby producing an intermediate product (Mishra & Baek, 2021).

## Conclusions

On a short note, using shotgun metagenomic sequencing, this study presents one of the foremost attempts to unveil the functional genes produced by the rhizobiomes of tomato plants’ rhizosphere and bulk soil. The PGP and disease-resistant genes were confirmed to be dominant in the HR, which showed how the microbial communities present in the HR contribute to the growth and health status of the tomato plants thereby improving the safety of the fruits produced and their sustainability. The abundance of these genes as obtained in this study revealed that the rhizosphere soil contains significant beneficial microorganisms that improve the health status and production of tomato fruits. Since not all of the microorganisms obtained are unculturable, further studies should be conducted on the existence of culturable ones. The microorganisms should be cultured and introduced in field experiments to investigate how these soil organisms produced functional metagenomes contributing to the growth of tomato plants. Our study, however, explained the influence of microorganisms present in the rhizosphere soil to promote growth, biological control potential, and improve the health status of the tomato plant. In the culture-independent approach, some bacterial genera were not revealed as obtained in the culture-dependent approach that revealed varieties of bacteria genera according to the data obtained by Pereira et al. (2011). Some challenges were encountered in trying to culture the unculturable organism. The culture-independent approach may be subdued by the heterogeneous lysis of various microbial species and may suffer from primer bias. Nevertheless, they purportedly supply complete data of the microbial communities encompassing viable but not culturable and also nonviable microorganisms.

## Supplemental Information

10.7717/peerj.15432/supp-1 Supplemental Information 1 Supplemental Tables.

## Additional Information and Declarations

### DNA Deposition

The following information was supplied regarding the deposition of DNA sequences: The raw sequence reads are available at NCBI BioProject: PRJNA766489.

### Data Availability

The following information was supplied regarding data availability: The raw data is available in the Supplemental Files.
