# Supplementary material for: Unraveling the functional genes present in rhizosphere microbiomes of Solanum lycopersicum
Source: PeerJ. 2023 Jun 2;11:e15432. doi: 10.7717/peerj.15432 (PMC10241170; doi:10.7717/peerj.15432)
Supplement: Supplemental Information 1 [file peerj-11-15432-s001.docx]

**SUPPLEMENTARY DATA**

**Table S1: Analysis of sequenced data and diversity evaluation of the shotgun metagenome from the rhizosphere and bulk soil of the tomato plant**

| **Sample site** | **HR** | **DR** | **BR** |  |
| --- | --- | --- | --- | --- |
|  | **Sequence uploaded** | | |  |
| **bp Count** | 2152004650.3 bp | 1409528303.3 bp | 1477197003 bp | |
| **Sequences Count** | 13739258.3 | 19765082 | 138145859.3 |  |
| **Mean Sequence Length** | 154±32 bp | 155±32 bp | 155±32 bp |  |
| **Mean GC percent** | 64±10 % | 64±10 % | 65±9 % |  |
| **Artificial Duplicate Reads** | 836435 | 757641.7 | 374748 |  |
|  | **Post QC sequences** | | |  |
| **bp Count** | 765041235.3 | 1352124415 | 1385218693 |  |
| **Sequences Count** | 12665143.7 | 11966279.7 | 5247459 |  |
| **Mean Sequence Length** | 155±33 bp | 156±39 bp | 156±34 bp |  |
| **Mean GC percent** | 63±9 % | 64±9 % | 64±9 % |  |
|  | **Processed Sequences** | | |  |
| **Predicted Protein Features** | 7850484.3 | 4208873.7 | 11448097.3 |  |
| **Predicted rRNA Features** | 28853 | 30027 | 25322.7 |  |
|  | **Alignment Sequences** | | |  |
| **Identified Protein Features** | 3985525.7 | 4178206.3 | 2515439.7 |  |
| **Identified rRNA Features** | 5962.7 | 7656.7 | 5439.3 |  |

Values presented in the table are the means of the replicates from each cropping site. Healthy rhizosphere (HR), diseased rhizosphere (DR) of the tomato plant, and bulk soil (BR)

**Table S2: Evaluation of major phyla in the rhizosphere of the tomato plant, and bulk soil.**

| **Phylum** | HR | DR | BR | p-value |
| --- | --- | --- | --- | --- |
| **Bacteroidetes** | 3.0±0.2 | 3.39±0.2 | 3.96±0.2 | 0.08 |
| **Planctomycetes** | 3.40±0.1 | 2.6±0.1 | 2.59±0.1 | 0.08 |
| **Proteobacteria** | 54.0±0.42 | 45.9±2.06 | 38.8±2.41 | 0.05 |
| **Chloroflexi** | 2.84±0.2 | 1.27±0.03 | 2.55±0.2 | 0.05 |
| **Firmicutes** | 2.43±0.1 | 1.69±0.06 | 2.31±0.2 | 0.07 |
| **Acidobacteria** | 5.18±0.6 | 2.28±0.1 | 5.0±0.2 | 0.07 |
| **Cyanobacteria** | 1.96 ± 0.3 | 0.92 ± 0.05 | 1.84 ± 0.23 | 0.06 |
| **Actinobacteria** | 35.50±2.3 | 25.36±0.6 | 28.13±0.6 | 0.03 |
| **Gemmatimonadetes** | 1.6 ± 0.07 | 1.31 ± 0.1 | 1.1 ± 0.03 | 0.11 |
| **Verrucomicrobia** | 2.34 ± 0.1 | 2.23 ± 0.10 | 2.15 ± 0.1 | 0.67 |
| **unclassified (derived Bacteria)** | 0.65 ± 0.01 | 0.9 ± 0.06 | 0.89 ± 0.02 | 0.06 |
| **Deinococcus-Thermus** | 0.43 ± 0.02 | 0.42 ± 0.02 | 0.28 ± 0.01 | 0.05 |
| **Spirochaetes** | 0.06 ± 0.003 | 0.07 ± 0.001 | 0.08 ± 0.001 | 0.06 |
| **Aquificae** | 0.06 ± 0.01 | 0.06 ± 0.003 | 0.04 ± 0.002 | 0.07 |
| **Synergistetes** | 0.05 ± 0.003 | 0.05±0.001 | 0.04 ± 0.002 | 0.06 |
| **Chlorobi** | 0.15 ± 0.01 | 0.12 ± 0.01 | 0.15 ± 0.01 | 0.07 |
| **Nitrospirae** | 0.39 ± 0.02 | 0.37 ± 0.03 | 0.31 ± 0.02 | 0.10 |
| **Thermotogae** | 0.05 ± 0.003 | 0.05±0.01 | 0.03 ± 0.001 | 0.07 |
| **Euryarchaeota (Archaea)** | 0.43 ± 0.03 | 0.41 ± 0.03 | 0.25 ± 0.02 | 0.05 |
| **Thaumarchaeota (Archaea)** | 0.29 ± 0.03 | 0.18 ± 0.04 | 0.15 ± 0.05 | 0.15 |
| **Crenarchaeota (Archae)** | 0.13 ± 0.01 | 0.06 ± 0.01 | 0.10 ± 0.01 | 0.06 |
| **unclassified (Archaea)** | 0.04 ± 0.002 | 0.03 ± 0.004 | 0.02 ± 0.001 | 0.06 |
| **Korarchaeota (Archaea)** | 0.003 ± 0.000 | 0.004 ± 0.000 | 0.002 ± 0.000 | 0.04 |
| **Nanoarchaeota (Archaea)** | 0.00 ± 0.000 | 0.00 ± 0.000 | 0.00 ± 0.000 | 0.07 |
| **Ascomycota (Fungi)** | 0.79 ± 0.11 | 0.45 ± 0.07 | 0.45 ± 0.05 | 0.07 |
| **Basidiomycota (Fungi)** | 0.03 ± 0.004 | 0.02 ± 0.001 | 0.01 ± 0.001 | 0.19 |
| **Blastocladiomycota (Fungi)** | 0.00±0.000 | 0.00±0.000 | 0.00±0.000 | 0.67 |

**Key:** Mean±Standard error. Healthy rhizosphere (HR), diseased rhizosphere (DR) of the tomato plant, and bulk soil (BR)

**Table 3: Plant growth-promoting genes in the rhizosphere soil of tomato and bulk soil**

| **Genes** | HR1 | HR2 | HR3 | DR1 | DR2 | DR3 | BR1 | BR2 | BR3 | p-value |
| --- | --- | --- | --- | --- | --- | --- | --- | --- | --- | --- |
| Indolepyruvate oxidoreductase subunit (*IorB*) | 0.004781 | 0.004044 | 0.00397 | 0.003333 | 0.003137 | 0.004245 | 0.003337 | 0.003248 | 0.004087 | 0.43 |
| Siderophore biosynthesis non-ribosomal peptide synthetase modules (*NRPS*) | 0.023151 | 0.023192 | 0.034325 | 0.010967 | 0.012101 | 0.012472 | 0.028338 | 0.025723 | 0.024121 | 0.06 |
| Ferric siderophore transport system, periplasmic binding protein (*TonB*) | 0.008766 | 0.006417 | 0.005333 | 0.006349 | 0.007583 | 0.00767 | 0.004894 | 0.00504 | 0.006131 | 0.11 |
| Ferric siderophore transport system, biopolymer transport protein (*ExbB*) | 0.003737 | 0.004704 | 0.005729 | 0.009713 | 0.008177 | 0.010059 | 0.006346 | 0.006139 | 0.005833 | 0.03 |
| Iron siderophore receptor protein (*iroN*) | 0.00034 | 0.001121 | 0.001009 | 0.000496 | 0.000403 | 0.001104 | 0.000445 | 0.000224 | 0.000235 | 0.19 |
| Iron siderophore sensor protein (*fecR*) | 0.001247 | 0.000791 | 0.00185 | 0.000523 | 0.000667 | 0.000704 | 0.000638 | 0.000403 | 0.000932 | 0.11 |
| 1-aminocyclopropane-1-carboxylate deaminase (*acdS*) | 0.012562 | 0.01198 | 0.009891 | 0.010175 | 0.010733 | 0.011516 | 0.010588 | 0.013291 | 0.011993 | 0.49 |
| Methylthioadenosine deaminase (*mtaD*) | 0.028714 | 0.033469 | 0.03558 | 0.022237 | 0.024868 | 0.022948 | 0.033187 | 0.030577 | 0.029448 | 0.06 |
| Nitrogenase (molybdenum-iron) reductase and maturation protein (n*ifH*) | 4.48E-05 | 0.000248 | 6.9E-05 | 0.000101 | 9.89E-05 | 0.000151 | 0.000222 | 7.47E-05 | 6.7E-05 | 0.73 |
| Two-component nitrogen fixation transcriptional regulator (*fixJ*) | 0.010023 | 0.008088 | 0.009453 | 0.004167 | 0.005531 | 0.006695 | 0.003337 | 0.005339 | 0.006968 | 0.07 |
| Tryptophan biosynthesis protein TrpCF (*trpC*) | 0.040878 | 0.040016 | 0.042485 | 0.040439 | 0.040657 | 0.044054 | 0.043008 | 0.042599 | 0.042893 | 0.25 |
| Indolepyruvate ferredoxin oxidoreductase, alpha and beta subunits (*iorAB*) | 0.088472 | 0.079697 | 0.083583 | 0.058747 | 0.051846 | 0.063434 | 0.046267 | 0.049244 | 0.058728 | 0.04 |
| Indolepyruvate oxidoreductase subunit (*iorA*) | 0.013908 | 0.015004 | 0.015793 | 0.010054 | 0.012395 | 0.012429 | 0.011913 | 0.013309 | 0.013632 | 0.05 |
| Indoleacetamide hydrolase (*iaaH*) | 0.008897 | 0.009035 | 0.009715 | 0.006349 | 0.006528 | 0.006997 | 0.007977 | 0.008514 | 0.008939 | 0.04 |
| Indole-3-pyruvate decarboxylase (*ipdC*) | 0.000945 | 0.001121 | 0.000875 | 0.000445 | 0.000448 | 0.000871 | 0.00039 | 0.000224 | 0.000104 | 0.03 |
| Catalase (*katE*) | 0.106965 | 0.105037 | 0.125729 | 0.146106 | 0.146139 | 0.14847 | 0.098761 | 0.101698 | 0.112966 | 0.05 |
| Tryptophan synthase alpha chain (*trpA*) | 0.047446 | 0.043537 | 0.044811 | 0.040151 | 0.043325 | 0.045039 | 0.043019 | 0.041366 | 0.044691 | 0.3 |
| Tryptophan synthase beta chain (*trpB*) | 0.109608 | 0.109819 | 0.113204 | 0.10393 | 0.105769 | 0.110323 | 0.119359 | 0.106514 | 0.113 | 0.3 |
| Pyoverdine chromophore precursor synthetase (*pvdL*) | 0.02687 | 0.028785 | 0.023853 | 0.023884 | 0.024538 | 0.024605 | 0.020786 | 0.020212 | 0.021093 | 0.25 |
| Pyoverdine sidechain non-ribosomal peptide synthetase *PvdD* | 0.018191 | 0.023089 | 0.00018 | 0.01312 | 0.013303 | 0.013848 | 0.019748 | 0.022047 | 0.017809 | 0.25 |
| Pyoverdine sidechain non-ribosomal peptide synthetase *PvdI* | 0.025279 | 0.000242 | 0.031319 | 0.014298 | 0.01431 | 0.015722 | 0.032085 | 0.024918 | 0.025695 | 0.60 |
| Pyoverdine sidechain non-ribosomal peptide synthetase (*pvdJ*) | 0.007946 | 0.008352 | 0.00804 | 0.012429 | 0.000128 | 0.013836 | 0.011523 | 0.018104 | 0.011941 | 0.19 |
| Ammonia monooxygenase structural gene (*amoA*) | 0.010188 | 0.009373 | 0.009891 | 0.014784 | 0.016497 | 0.019896 | 0.020064 | 0.000163 | 0.015478 | 0.15 |
| adenylyl-sulfate kinase (*cysC*) | 0.001957 | 0.002144 | 1.83E-05 | 0.002071 | 0.002509 | 0.002765 | 0.001776 | 0.003 | 0.001749 | 0.06 |
| sulfate adenylyltransferase subunit 2 (*cysD*) | 0.287638 | 0.272235 | 0.2893 | 0.285896 | 0.286241 | 0.286559 | 0.284851 | 0.002752 | 0.28543 | 0.20 |
| Cold shock protein (*cspB*) | 0.095545 | 0.090353 | 0.000903 | 0.095617 | 0.096976 | 0.098671 | 0.094804 | 0.099301 | 0.091458 | 0.10 |
| Sulfate and thiosulfate binding protein (*cysP*) | 0.038403 | 0.035582 | 0.032299 | 0.030782 | 0.030993 | 0.031935 | 0.038037 | 0.000379 | 0.032664 | 0.25 |
| Sulfate transport system permease protein (*cysW*) | 0.000369 | 0.036315 | 0.037948 | 0.037262 | 0.038965 | 0.039434 | 0.031746 | 0.032444 | 0.030513 | 0.04 |
| Sulfate adenyltransferase subunit1 (*cysN*) | 0.047047 | 0.041943 | 0.044245 | 0.053136 | 0.054007 | 0.05746 | 0.043864 | 0.000434 | 0.045227 | 0.08 |
| Glutathione-regulated potassium-efflux system protein (*kefB*) | 0.031519 | 0.036445 | 0.023481 | 0.045351 | 0.049689 | 0.050578 | 0.01953 | 0.000192 | 0.029548 | 0.07 |
| Glutathione-regulated potassium-efflux system protein (*kefC*) | 0.016734 | 0.013219 | 0.015945 | 0.015596 | 0.01746 | 0.017931 | 0.010855 | 0.000112 | 0.01454 | 0.06 |
| Kup system potassium uptake protein (*kup*) | 0.116734 | 0.117958 | 0.001109 | 0.109333 | 0.111219 | 0.118631 | 0.122848 | 0.119914 | 0.120898 | 0.04 |
| Potassium uptake protein, integral membrane component, (*ktrA*) | 0.003589 | 0.003092 | 0.002313 | 3.17E-05 | 0.006425 | 0.004037 | 0.004574 | 0.004781 | 0.00335 | 0.05 |
| Potassium uptake protein, integral membrane component, (*ktrB*) | 0.010658 | 0.011408 | 0.008915 | 6.46E-05 | 0.005427 | 0.006935 | 0.00819 | 0.005691 | 0.008801 | 0.02 |

**Key:** Mean of the data. Healthy rhizosphere (HR 1-3), diseased rhizosphere (DR 1-3) of the tomato plant, and bulk soil (BR 1-3)

**Table 4: Disease resistant genes in the rhizosphere soil of tomato and bulk soil**

| **Genes** | HR1 | HR2 | HR3 | DR1 | DR2 | DR3 | BR1 | BR2 | BR3 | p-value |
| --- | --- | --- | --- | --- | --- | --- | --- | --- | --- | --- |
| Phosphate transport ATP-binding protein (*PstB*) | 0.116822 | 0.104412 | 0.104608 | 0.106903 | 0.103217 | 0.107336 | 0.085264 | 0.091194 | 0.083229 | 0.50 |
| Nicotinate-nucleotide adenylyltransferase (*nadD*) | 0.026274 | 0.024036 | 0.021957 | 0.019 | 0.022371 | 0.020949 | 0.022613 | 0.021392 | 0.020064 | 0.18 |
| Phosphate transport system permease protein (*PstA*) | 0.011097 | 0.009974 | 0.006946 | 0.012094 | 0.012414 | 0.012101 | 0.008631 | 0.008064 | 0.010151 | 0.15 |
| 2',3'-cyclic-nucleotide 2'-phosphodiesterase (*cpdB*) | 0.000623 | 0.000859 | 0.000737 | 0.000851 | 0.000483 | 0.000538 | 0.000495 | 0.000718 | 0.000404 | 0.07 |
| Dipeptide transport system permease protein (TC 3.A.1.5.2) (*dppB*) | 0.000437 | 0.001253 | 0.001398 | 0.001205 | 0.001104 | 0.000627 | 0.000934 | 1.34E-05 | 0.000905 | 0.03 |
| Nicotinamide-nucleotide adenylyltransferase (*nadM*) | 0.000461 | 0.001104 | 8.96E-05 | 0.000908 | 0.000528 | 0.000567 | 0.000302 | 0.000178 | 3.73E-05 | 0.25 |
| Ribonucleotide reduction protein (*NrdI*) | 3.55E-05 | 0 | 0 | 0 | 3.3E-05 | 0 | 0 | 3.73E-07 | 0.000235 | 0.14 |
| Anaerobic ribonucleoside-triphosphate (*nrdD*) | 0.08672 | 0.088318 | 0.094865 | 0.077248 | 0.075996 | 0.077417 | 0.086216 | 0.084289 | 0.00091 | 0.84 |
| Bacitracin export permease protein (*bceB*) | 0.127084 | 0.114835 | 0.124936 | 0.107316 | 0.100134 | 0.110619 | 0.118527 | 0.121939 | 0.121858 | 0.36 |
| Pyochelin synthetase, non-ribosomal peptide synthetase module (*pchF*) | 0.069348 | 0.066231 | 0.070302 | 0.062736 | 0.063008 | 0.060689 | 0.067265 | 0.065111 | 0.066031 | 0.05 |
| High-affinity branched-chain amino acid transport system permease protein (TC 3.A.1.4.1) (*livH*) | 0.072078 | 0.075014 | 0.07189 | 0.071239 | 0.067163 | 0.062809 | 0.069222 | 0.069404 | 0.070085 | 0.06 |
| Dipeptide transport system permease protein (TC 3.A.1.5.2) (*dppC*) | 0.029923 | 0.032398 | 0.032131 | 0.042781 | 0.040423 | 0.040908 | 0.027893 | 0.025499 | 0.034975 | 0.06 |
| Phosphate transport system permease protein (*pstC*) | 0.064061 | 0.000614 | 0.060101 | 0.052229 | 0.044083 | 0.044575 | 0.055734 | 0.061167 | 0.0595 | 0.06 |
| Glycerol-3-phosphate ABC transporter, permease protein (*ugpA*) | 0.133307 | 0.140124 | 0.140225 | 0.129931 | 0.130831 | 0.130866 | 0.123452 | 0.130968 | 0.129281 | 0.06 |
| Nucleoside-diphosphate-sugar epimerases (*wcaG*) | 0.007785 | 0.007467 | 0.007571 | 0.006063 | 0.00596 | 0.005695 | 0.005026 | 0.004616 | 0.005181 | 0.04 |
| iron aquisition yersiniabactin synthesis enzyme (*Irp1*, polyketide synthetase) | 0.000886 | 0.001031 | 0.000449 | 0.000416 | 0.000264 | 0.000303 | 0.00089 | 0.000597 | 0.000737 | 0.03 |
| Dihydroaeruginoate synthetase (*pchE*), non-ribosomal peptide synthetase modules | 0.000491 | 0.000528 | 0.000404 | 0.000578 | 5.23E-06 | 0.000402 | 0.000461 | 0.000538 | 0.000483 | 0.07 |
| Maltose/maltodextrin ABC transporter 2, permease protein (*MalG*) | 0.064284 | 0.063655 | 0.059431 | 0.054724 | 0.053414 | 0.053389 | 0.05974 | 0.056327 | 0.057567 | 0.05 |

**Key:** Mean of the data. Healthy rhizosphere (HR 1-3), diseased rhizosphere (DR 1-3) of the tomato plant, and bulk soil (BR 1-3)
